# The myths surrounding mild stimulation in vitro fertilization (IVF)

**DOI:** 10.1186/s12958-017-0266-1

**Published:** 2017-06-24

**Authors:** Raoul Orvieto, Valeria Stella Vanni, Norbert Gleicher

**Affiliations:** 1 Infertility and IVF unit, Department of Obstetrics and Gynecology, Chaim Sheba Medical Center, Tel Hashomer, Ramat Gan, Israel; 2 The Tarnesby-Tarnowski Chair for Family Planning and Fertility Regulation, Sackler Faculty of Medicine, Tel-Aviv University, Tel-Aviv, Israel; 3 Centro Scienze Natalità, Department of Obstetrics and Gynecology, IRCCS Ospedale San Raffaele, Milano, Italy; 4 The Center for Human Reproduction, New York, N.Y 10021 USA; 5 Foundation for Reproductive Medicine, New York, N.Y 10022 USA; 6 Laboratory of Stem Cell Biology and Molecular Embryology, The Rockefeller University, New York, N.Y 10065 USA; 7 Department of Obstetrics and Gynecology, University of Vienna School of Medicine, 1090 Vienna, Austria

**Keywords:** Ovarian stimulation, Aneuploidy, Cost-effectiveness, Ovarian hyperstimulation syndrome (OHSS), In vitro fertilization (IVF), Pregnancy rates, Live birth rates

## Abstract

So-called mild controlled ovarian hyperstimulation (mCOH) has in recent years increased in popularity, claiming to be safer and more patient-friendly, while also improving in vitro fertilization (IVF) outcomes. We here challenge the International Society for Mild Approaches in Assisted Reproduction (ISMAAR) definition of mild stimulation, and especially address four fundamental issues, where our review found conventional COH (cCOH) advantageous over mCOH. They are: prevalence of severe ovarian hyperstimulation syndrome (OHSS), oocyte/embryo quality, pregnancy/live birth rates, and cost. We conclude that an objective review of the literature does not support the routine utilization of mCOH in assisted reproduction.

## Background

Controlled ovarian hyperstimulation (COH) is essential for in vitro fertilization (IVF) success since it facilitates recruitment of multiple oocytes and, thereby, also often allows for multiple embryo transfers (ETs) [1], without causing ovarian hyperstimulation syndrome (OHSS). In recent years, milder approaches to COH have become increasingly popular, aiming to offer safer and more patient-friendly approaches to reduce patient stress, complications such as OHSS, and cost of IVF cycles [2, 3], while allegedly improving endometrial receptivity, embryo quality and implantation potential [4].

Since investigators used various different definitions and terminologies to describe mild controlled ovarian hyperstimulation (mCOH), the Rotterdam-based International Society for Mild Approaches in Assisted Reproduction (ISMAAR) Consensus Group on Terminology for Ovarian Stimulation for IVF, in 2007, proposed a concise and simplified revised nomenclature for different approaches to ovarian stimulation for IVF [5].

The term natural or unstimulated cycle IVF was proposed when IVF is carried out during a spontaneous menstrual cycle without gonadotropin support, aiming to collect the naturally selected single oocyte of the cycle. mCOH was defined as follicle stimulating hormone (FSH) or human menopausal gonadotropin (HMG) administered at lower doses, and/or for a shorter duration in a GnRH antagonist co-treated cycle, or when oral compounds (anti-estrogens, or aromatase inhibitors) are used, either alone or in combination with gonadotropins, aiming for collection of 2–7 oocytes. cCOH was defined by use of GnRH analogues (long/flare agonist or antagonist protocols) with conventional doses of stimulation with FSH or HMG, aiming to collect eight or more oocytes.

These definitions can, however, be quite confusing. For example, offering mCOH with a daily gonadotropin dose <150 IU, some patients may respond more vigorously than expected, yielding more than 8 oocytes. Such an oocyte count, however, is already in the cCOH range defined by ISMAAR. How, then, should such a cycle be defined, According to stimulation (mCOH), or patient response (cCOH)?

Four other fundamental issues require review:

### Prevalence of severe OHSS

Best IVF is characterized by balance between optimal ovarian stimulation with successful treatment outcomes and minimal rates of severe OHSS and/or (especially higher order) multiple pregnancies. Individualization of treatments, based on patient risk factors, cycle responses, elective single embryo transfer and the option of freezing all embryos have in susceptible cases the potential of reducing the risks and severity of the syndrome [6]. Withholding ovulation-inducing triggers of hCG, or replacing hCG with GnRH agonist (GnRHa) triggers may further reduce severe early OHSS. Especially GnRHa trigger, combining GnRH antagonist co-treatment and GnRHa trigger, has recently been increasinly utilized to eliminate severe early OHSS in support the OHSS-free clinic [7, 8].

Significantly reduced clinical pregnancies and increased first trimester pregnancy loss with such treatments [9, 10], however, popularized efforts to improve reproductive outcome through all-freeze policies, fresh transfers with intensive luteal support and fresh transfers with low-dose hCG supplementation. We previously published a comprehensive review of suggested strategies, to which the reader is referred [6]. It addresses the role of GnRH-antagonists in COH protocols, the use of different luteal rescue protocols, the role of cabergoline and the ability to transfer embryos at blastocyst stage.

Utilizing this knowledge, severe OHSS is no longer a complication to fear, as we practically, have eliminated it from our practice for over a decade.

### Oocyte/embryo quality

A study by Baart et al. [11] raised concerns after demonstrating lower embryo aneuploidy following mCOH compared to cCOH. While mCOH produced significantly fewer oocytes and embryos, both regimens generated similar numbers of chromosomally normal embryos. Bart et al., therefore, concluded that differences in aneuploid embryos suggests that mitotic segregation errors may increase with growing gonadotropin dosages. On the contrary, several other studies have disputed the aforementioned observation, demonstrating a comparable aneuploidy rate in stimulated and un-stimulated cycles.

In 2012, Labarta et al. [12] compared unstimulated and stimulated IVF cycles in the same young (<35 yrs) egg donors. Using in those days preimplantation genetic screening (PGS) with fluorescence in situ hybridization (FISH) on cleavage-stage embryos, these authors reported similar aneuploidy rates in unstimulated and stimulated cycle (34.8% and 40.6%, respectively), with no in-between group differences in embryo quality and types of chromosomal abnormalities. These aneuploidy rates concurred with 36.4% rates already in 2009 reported by Verpoest et al. in unstimulated cycles [13]. Also in 2012, using similar early-stage PGS technologies, Gleicher et al. [14] in normally fertile women undergoing IVF for sex selection purposes, reported mild increases in aneuploidy with increasing gonadotropin dosages; but those increases were more than compensated for by larger numbers of transferrable euploid embryos in women who received higher gonadotropin stimulation because of larger oocyte yields. Already in1992, Gras et al. [15] reported that the rate of aneuploidy in non-stimulated oocytes was comparable to oocytes obtained after COH with either clomiphene/hMG or the flare GnRHa protocol.

Like Labarta et al. [12] and Gleicher et al. [14], Baart et al. [11] used FISH to determine copy number of only nine chromosome pairs of cleavage-stage embryos on day-3 after fertilization, a method since discarded due to high imprecision [16, 17]. Aneuploidy rates reported by Baart et al. (66% and 67% percent, respectively) were also much higher than those by Labarta et al. [12] and Verpoest et al. [13] (34.8% and 36.4%, respectively). They also compared the aneuploidy rates, comparing presumed aneuploid and mosaic embryos when analyzing two blastomers. As we now know, mosaicism is much more common than previously appreciated and, likely, a normal physiological phenomenon of developing embryos with, still, strong innate abilities to self-correct downstream beyond blastocyst stage [reviewed in [17]]. This was best demonstrated by the achievement of surprisingly high normal live birth rates after transfer of mosaic/aneuploid embryos [18–20]. Adding, however, up rates of normal euploid and mosaic embryos in the study by Baart et al. [11], the percentages of clinically likely normal embryos (28+33=61% v.s 39+21=60%), in mCOH and cCOH groups, suddenly, no longer differs.

A recent study [21] of patients undergoing IVF cycles involving COH and trophectoderm biopsy for PGS has demonstrated that the degree of exposure to exogenous gonadotropins did not significantly modify the likelihood of aneuploidy in patients with a normal ovarian response to stimulation (not requiring COH beyond cycle day 12). Patients requiring prolonged COH were demonstrated to have elevated odds of aneuploidy with increasing cumulative gonadotropin dose. Finding that may reflect an increased tendency towards oocyte and embryonic aneuploidy in patients with a diminished response to gonadotropin stimulation.

We, therefore, conclude that there is no reliable evidence to suggest that more aggressive ovarian stimulation increases aneuploidy rates in embryos to significant degrees.

### Pregnancy/live birth rates

Several studies have dealt with pregnancy\live birth rates in mCOH vs cCOH, demonstrating a huge heterogeneity in terms of inclusion criteria, numbers of oocyte retrieved/embryo transferred, or day of the transfer. Verberg et al. [22] conducted a meta-analysis, aiming to investigate whether retrieval of low numbers of oocytes following mCOH is associated with impaired implantation rates. Including three randomized controlled trials of a total of 592 first treatment cycles, optimal embryo implantation rates were observed with retrieved 5 oocytes following mCOH (31%), versus 10 oocytes following cCOH (29%). These finding suggest that modest numbers of oocytes following mCOH are not a reflection of poor ovarian response.

Distribution of retrieved oocytes was similar for both protocols (range 0–28), with median (25–75 percentile) of 6 [3–10] and 9 [6–12] following mCOH and cCOH, respectively. Overlapping results made it also impossible, to categorized patients to mCOH or cCOH, based on the definition of the Rotterdam ISMAAR Consensus group [5]. Moreover, when comparing ongoing pregnancy rates (PR) per fresh transfer, they were significantly higher in the cCOH vs. mCOH (29% vs 15%; *p* < 0.001, respectively).

Since mCOH cycles more frequently than cCOH cycles fail to reach embryo transfer, outcome analysis with reference cycle start (“intent to treat”) would, likely, demonstrate even more impressive differences in favor of cCOH.

More impressive difference was also found comparing stimulated to unstimulated cycles. Sunkara et al. [23] recently analyzed anonymous data obtained from the Human Fertilisation and Embryology Authority (HFEA), involving 584,835 stimulated IVF cycles and 6168 unstimulated IVF cycles. The overall live birth rates were 4.7% per cycle following unstimulated fresh IVF versus 22.5% following stimulated fresh IVF. In other words, 3.5 times as many unstimulated IVF cycles are required to achieve one live birth compared to stimulated IVF.

Above noted advantages of cCOH were demonstrated in first fresh-cycle transfers. Those advantages would also become even more obvious if additional frozen-thawed cycle were to be included. Moreover, optimal embryo implantation rates observed with 5 oocytes following mCOH [22] are really irrelevant because they fall far below the required oocyte yields for a live birth, reported to be 14–15 metaphase II oocytes, 10 day-2 or day-3 embryos or 5 blastocysts in 70% of patients [24, 25].

It was recently also demonstrated [26], that the cumulative live birth rate (LBR) following the transfer of all fresh and frozen–thawed embryos after a single ovarian stimulation, significantly increases with the number of oocytes retrieved. High responders (>15 oocytes) demonstrated a significantly higher LBR not only versus poor (0–3 oocytes) and suboptimal [4–9] responders, but also versus women with normal [10–15] ovarian response. While suboptimal responders had a better outcome compared with poor ovarian responders, this group had a significantly lower cumulative LBR compared with normal ovarian responders [26].

### Cost

Groen et al. [27] evaluated the cost-effectiveness of modified natural cycle (MNC) versus cCOH. MNC was not cost-effective, as conventional COH dominated MNC with a higher cumulative LBR and lower cost per patient. LBR per cycle was 3.8 higher in the conventional vs. MNC COH (23% and 6%, respectively), while the cost was 1.8 higher (2110 vs 1150 Euro. Extrapolating the data to mCOH, which involves more medication (gonadotropins), and taking into consideration the total reproductive potential of each initiated IVF cycle (i.e. fresh plus subsequent frozen/thawed transfers) with reference point cycle start (i.e.,” intent to treat”) [25], cCOH would be advantageous in term of cost-effectiveness per cumulative LBR.

## Conclusion

mCOH has been proposed to provide safer and more patient-friendly IVF, with improving outcomes. Upon careful review, it offers none of these advantages. Regarding occurrence of severe OHSS, oocyte/embryo quality, pregnancy/live birth rates and cost, cCOH is at least comparable or sometime superior over mCOH, discrediting the concept of using mCOH in routine IVF. Further large prospective studies are needed to compare and clarify the role of mCOH vs cCOH in the different subgroups of patients. Moreover, these studies may help fertility specialists in individualization and careful tailoring of the COH protocol for optimizing IVF success.

## Abbreviations

cCOH: Conventional controlled ovarian hyperstimulation
COH: Controlled ovarian hyperstimulation
ET: embryo transfers
FISH: fluorescence in situ hybridization
GnRHa: GnRH agonist
HFEA: Human Fertilisation and Embryology Authority
HMG: human menopausal gonadotropin
ISMAAR: International Society for Mild Approaches in Assisted Reproduction
IVF: in vitro fertilization
mCOH: mild controlled ovarian hyperstimulation
MNC: modified natural cycle
OHSS: ovarian hyperstimulation syndrome
PGS: preimplantation genetic screening
